# Get to Understand More from Single-Cells: Current Studies of Microfluidic-Based Techniques for Single-Cell Analysis

**DOI:** 10.3390/ijms160816763

**Published:** 2015-07-23

**Authors:** Shih-Jie Lo, Da-Jeng Yao

**Affiliations:** Institute of Nanoengineering and Microsystem, National Tsing Hua University, Hsinchu 300, Taiwan; E-Mail: roxyjay0406@gmail.com

**Keywords:** microfluidic techniques, single-cell manipulation, single-cell analysis

## Abstract

This review describes the microfluidic techniques developed for the analysis of a single cell. The characteristics of microfluidic (e.g., little sample amount required, high-throughput performance) make this tool suitable to answer and to solve biological questions of interest about a single cell. This review aims to introduce microfluidic related techniques for the isolation, trapping and manipulation of a single cell. The major approaches for detection in single-cell analysis are introduced; the applications of single-cell analysis are then summarized. The review concludes with discussions of the future directions and opportunities of microfluidic systems applied in analysis of a single cell.

## 1. Introduction

A single cell is the smallest functional unit within an organism and maintains the functions of tissues through mutual cooperation. Biological research has extended from cell physiology and mechanics to gene expression. Accompanied by numerous achievements of biological research over hundreds of years, biology has progressed to becoming a data-rich subject; *omic biology* thus is integral to large-scale study (*i.e.*, genome, proteome, connectome *etc.*). Apart from the rise of omic biology, in single-cell biology there have been investigations of the effect of one or a few genes in thousands of single cells over a long period of time. Single-cell biology utilizes speed-limited approaches (*i.e.*, fluorescence *in situ* hybridization; FISH) to study functions in a single cell, whereas omic biology employs high-throughput approaches (*i.e.*, whole genome sequencing) to collect much data rapidly. Note, omic biology passes over variations between cells. In recent years, molecular biotechnology and the related techniques have progressed toward quantitative analysis of a single cell. In this way, omic biology moves in the same direction as single-cell biology, namely studying many genes in many single cells [1]. No matter in which direction biological research evolves, single-cell analysis hence remains an important and fundamental topic in the biological field.

Although analysis of a single-cell plays an important role in biological search for an understanding of functions in an organism, analytical techniques face diverse challenges: (1) the analytical tool should work well with a single cell as the size of a cell is below the micrometer scale, and the target in a single cell of interest is thus on a sub-micrometer or even nanometer scale; (2) the analysis is conducted using little sample; (3) the analytical approach could be handled with high throughput so as to acquire sufficient data for significant statistics; and (4) the analytical tool is universally affordable for laboratories. Microfluidic systems thus conform to these requirements and have exhibited great performance in recent decades [2]. Microfluidic systems transport and manipulate a liquid volume ranging from microlitres to femtolitres in microfluidic channels of which the dimensions are on a micrometer scale. A living biological specimen has typically a liquid form; as a result microfluidics is favorable for single-cell analysis. Moreover, microfluidic chips are made of inexpensive materials; the chips can certainly be designed for high-throughput use. Microfluidic chips have hitherto been fabricated from silicon or glass, elastomer, thermosets, hydrogel, thermoplastics and paper [3]. Microfluidic chips are sometimes constituted as multiple layers of varied materials for a specific purpose [4,5], *i.e.*, an elastomer bound to glass for a product with increased rigidity. Polydimethylsiloxane (PDMS) is an elastomer of one kind that has become a popular material for the fabrication of microfluidic chips because of its low cost, gas permeability and biocompatibility; most microfluidic devices related to biological research are consequently made of PDMS. In this review, we hence focus on the microfluidic-based techniques that have been applied in single-cell analysis and highlight some new developments.

## 2. Isolation, Trapping and Manipulation of Single Cells

**Figure 1:**
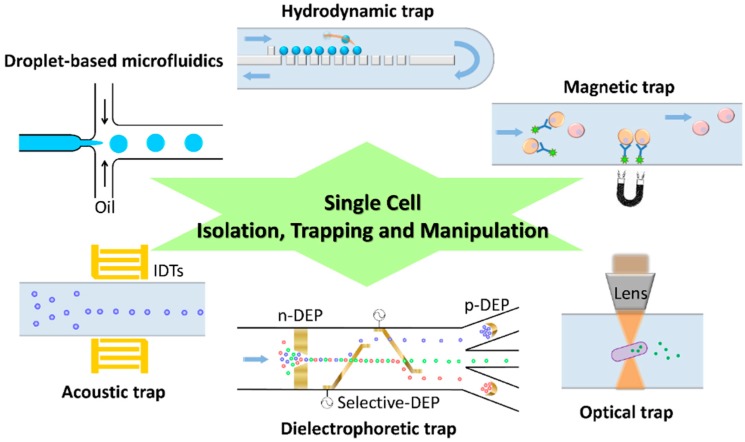
Schematic diagram of diverse approaches for the isolation, trapping and manipulation of single cells in a microfluidic device.

Before initiating a single-cell analysis, cells of interest must be selected for further treatment. One advantage of a microfluidic device is that one can generate droplets with tiny volumes (nL to fL) as a separated environment for growth or chemical reaction of a single cell. Table 1 summarizes the microfluidic techniques for single-cell analysis and Figure 1 shows an overview of various approaches for the isolation, trapping and manipulation of a single cell. The concept of droplet-based analysis is similar to that of micro-wells, significantly decreasing the possibility of cross-contamination. The generated droplets are then selected with a flow cytometer. Flow cytometry is a historic and commonly used technique in biological laboratories for high-throughput sorting of cells of interest; it can be used for analysis of the cell properties according to various parameters, *i.e.*, cell morphology, cell viability, protein expression *etc*. Accompanying the development of microfluids, flow cytometry is unrestricted to a cumbersome instrument, and can be operated in a pocket chip. The selection of cells of interest in a microfluidic-based flow cytometer can be classified into optical sorting and magnetic sorting. In optical sorting, the design of a flow cytometer is generally constituted by switching valves with a branched microfluidic channel. The switching valve can be mechanically based or bubble-based [6]. The width and height of a fluidic channel are sufficient for a single cell but not two cells concurrently passing through. The fluorescent signal of each cell is the selection criterion of optical sorting, which is excited by a microfluidic integrated laser system and emitted from fluorescein-linked antibodies or fluorescent probes; the cells of interest become guided to the reservoir via the function of a switching valve. This optical sorting is also named fluorescence-activated cell sorting (FACS) [7]. The cell selection of interest might thus be achieved with a flow cytometer via a specific antibody–antigen conjugation and labeling with a fluorescent probe. Secondly, according to the magnetic sorting approach, magnetic-affinity cell sorting (MACS) [8], also employs the specificity of an antibody–antigen conjugation, but the difference is a magnetic-bead-linked antibody rather than a fluorescein-linked one. To select a cell in magnetic sorting involves applying a magnetic field at a cell-collection reservoir, through which a fluid flows, to capture the magnetic-bead-labeled cells. A droplet-based microfluidic device with the function of flow cytometry can thus achieve single-cell analysis of various types [9]. A droplet-based microfluidic device can also be integrated with various sorting techniques, introduced in what follows, to select the target cells or compounds for analysis.

To date, varied trap techniques are commonly utilized and combined with microfluidic systems for observation or manipulation. If enduring manipulation is required, the trapped single cells also can be cultivated on a chip with nutrition supplied in a continuous flow. In a flow cytometer, the cells of interest are selected. In what follows we introduce the commonly used techniques of cell separation and manipulation for advanced analysis of single cells.

**Table 1:** Comparison of various microfluidic techniques for single-cell analysis.

Approaches	Main Applications	Major Advantage	Major Disadvantage
**Droplet-based microfluidics**	Single cell isolation	High throughput screening for specific single-cells	Challenge to encapsulate single-cells in each droplet
	Cell culture in droplet		
**Hydrodynamic trap**	Single cell isolation	Multifunction in one device	Complicated fabricating process
	Cell culture on chip		
**Magnetic trap**	Specific cell trapping	Efficient trapping of labeled cells	Requires antibodies or primers for magnetic label
**Acoustic trap**	Cell manipulation	Good for cell positioning	May have negative effect to cells
**Dielectrophoretic trap**	Cell manipulation	Easily select target cells via alternating frequency of AC	Heat problem during long-term manipulation
	Cell selection		
**Optical trap**	Cell manipulation	Applicable in many fields of study	Requires expensive optical system
	Cell mechanics		

### 2.1. Hydrodynamic Trap

Hydrodynamic trap utilizes specific microstructures and valves in a microfluidic channel to control the fluid flow so as to collect single cells without the use of other apparatus. This method is simple, but the fabrication of a microfluidic channel might be complicated because of microstructures. The structure of a microfluidic channel acts like a filter to trap cells within the fluid flow so as to acquire many single cells, but the number of cells lost is considerable, which should be considered in applications. Long *et al.* [10] fabricated a microfluidic chip containing two large feeding channels connected with multiple trapping or growth channels on a sub-micrometer scale. This ladder-like microfluidic chip was used to study the population of *E. coli* responding to dynamic changes in their environment that was achieved on varying the composition of growth media in feeding channels. Lin *et al.* [11] demonstrated sieve-like trap arrays in a microfluidic channel to trap and to position single cells on a glass substrate for their interactive study. Various paired configurations to trap cells were efficiently investigated and discussed in this work, providing an alternative approach for cell patterning. Secondly, there is another kind of hydrodynamic trap which employs the characteristics of fluidics via alternating the flow rate, causing either laminar flows or vortex flows, so as to achieve a specific purpose such as locating targets at the desired micro-structure. Sochol *et al.* [12] demonstrated a resettable hydrodynamic arraying system for trapping and releasing the target single cells. Although the performance of target trapping is important, the efficiency of target releasing is also a major concern in device development. In their work, the loading efficiency of the device was finally 99.8% and 78% for bead-based and cell-based experiments, accordingly. Wang *et al.* [13] developed a microfluidic hydrodynamic trapping system with the capability of long-term monitoring the cellular dynamics. The microfluidic device has a special bypass structure, which alternates the hydrodynamics in flow channel, and traps single-cells at the desired locations. The microfluidic trapping array has single cell trapping efficiency of ~90% and used as a tool for evaluating the efficiency of chemotherapeutic reagents.

### 2.2. Optical Trap

Optical trap is also called *optical tweezers*, which is a highly precise technique for manipulating micro-scale objects. The optical trap utilizes laser beam to generate a force sub-pN in a microfluidic channel [14], which is sufficient to trap single cells. From a combination of an optical system and a microfluidic system, a new research field called optofluidics has emerged. On altering the focus of laser beams in a microfluidic channel, an object (*i.e.*, blood cells, PS beads, *etc.*) can be manipulated according to its shape, size and refractive index. The cost of using an optical trap to separate single cells is great because of the optically controlled system to locate precisely the focus of laser beams. Moreover, prolonged manipulation in an optical trap might cause a problem because the cells become damaged through the heat generated from the laser energy. Liberale *et al.* [15] developed an integrated microfluidic device containing a micro-prism structure, which was fabricated with two-photon photolithography and allowed light from an optical fiber to trap a single cell. The integrated microfluidic device is capable of on-chip manipulation, Raman and fluorescence spectra of single cells. An optical trap has been developed to alter the shape of an aperture to improve the trapping efficiency, such as a rectangle, a double nanohole (DNH) and a coaxial aperture. The DNH optical trap has been utilized to study protein–protein interaction [16] and protein–DNA interaction [17], and also to determine the size and concentration of nanoparticles in solution [18].

### 2.3. Magnetic Trap

The isolating technique based on magnetic force functions through an action of immunomagnetic labeling or a hybridization of a nucleic-acid probe modified with magnetic beads. The objects of interest contain antigens that can be recognized by specific antibodies; the antibodies are linked with dextran-coated magnetic particles. The magnetically labeled objects can hence be captured in a microfluidic device treated with a magnetic field. The separation can be implemented through positive selection (*i.e.*, collect objects linked with magnetic beads) or negative selection (*i.e.*, collect objects without magnetic beads linked). The only disadvantage is the specificity of a magnetic trap depending on the antibodies and the primer design. Lai *et al.* [19] developed a microfluidic chip integrated with a magnetic trap for the screening of aptamers specific to influenza A virus; the aptamer screening, also called systematic evolution of ligands and exponential enrichment (SELEX), was shortened to 60 min with this micro fluidic chip, to be compared with a conventional process that requires at least 160 min. Chen *et al.* [20] developed a mobile magnetic trap array, which was integrated with a droplet-generating microfluidic device, to encapsulate magnetically selected single cells as a powerful analytical tool for a single cell. Nawarathna *et al.* [21] developed an integrated nanoscale magnetic trap within a plastic microfluidic device; the magnetic field gradients therein were significantly increased to trap magnetic beads efficiently.

### 2.4. Dielectrophoretic Trap

Dielectrophoresis (DEP) is a phenomenon that involves a motion of polarizable particles under a non-uniform electric field. The types of DEP can be briefly classified into positive DEP (p-DEP) and negative DEP (n-DEP) [22,23], depending on the permittivity of the polarizable particles and the surrounding medium. When the permittivity of particles is greater than that of the medium, the particles have polarized opposite charges in the electric field; the particles move to the direction of a strong electric field, which is called p-DEP. n-DEP exhibits conversely that the particles move to the direction of a weak electric field when the permittivity of particles is less than that of a medium. The particles have polarized identical charges in the electric field because the polarized charges of the particles surface become greater than that of the particle inside. The electrical property of a particles surface and the interior is opposite as a result of the particles being induced with identical charges in the electric field. DEP is readily incorporated into a microfluidic device via a fabricated microelectrode. Cell patterning in microfluidic device is achievable via a DEP approach with an electrode array [24]. A dielectrophoretic trap has also a smaller heat problem than an optical trap during a protracted manipulation, because the heat generated is less and is removed adequately with the fluid flow. Bhattacharya *et al.* [25] employed insulator-based dielectrophoresis (iDEP) to trap single mammalian breast-cancer cells (MCF-7) from mixtures with mammalian peripheral blood mononuclear cells (PBMC). Simulation and experimental results of their tear-drop structure in a microfluidic channel indicate the weakly metastatic cancers cell (MCF-7) could also be selectively trapped from a mixture containing the highly invasive cancer cells (MDA-MB-231), so that this iDEP based microfluidic device might be applied as a diagnostic tool for breast cancer. Huang *et al.* [26] also used a DEP-integrated microfluidic device for single MCF-7 cell trapping, and investigated the effects on a trapped cell under a varied applied electric field, suggesting that an applied electric field less than 2 kV/cm was safe for cell viability. An alternative material, a carbon electrode, has been used as a function of electrode and insulator structure for DEP applications [27].

### 2.5. Acoustic Trap

A surface-acoustic wave can be used as a non-contact approach to sense a specific analyte or to confine single cells in a microfluidic channel. The principle of an acoustic trap is that an ultrasonic standing wave, produced with a pair of interdigital transducers (IDT) while applying electricity, traps or agglomerates cells. The IDT structure is deposited on a piezoelectric substrate in a parallel or orthogonal arrangement for varied cell patterning [28]. The negative effect of acoustic manipulation has been an issue and discussed [29,30], but little evidence verifies that exposure to ultrasound decreases the survival rate of cells. Chen *et al.* [31] developed a microfluidic device based on a standing surface-acoustic wave (SSAW) for continuous enrichment of a limited cell sample. The limited sample can be concentrated within the standing waves in a microfluidic channel and be eventually collected for further cellular study. The systems of acoustic trap and optical tweezers are currently combined as optoacoustic tweezers in a microfluidic device with the characteristics of biocompatibility and ease of fabrication for dynamically concentrating and patterning particles and cells [32].

## 3. Single-Cell Analysis in a Microfluidic Device

As varied techniques to trap a single are applicable in a microfluidic device, techniques of microanalysis are equally important in the analysis of a single cell. Electrophoresis is a commonly used technique to separate small molecules via varied electrophoretic mobility of charged molecules in an electric field. A major difference between electrophoresis and dielectrophoresis (DEP) depends on the objects being charged, or not. The separation principle of electrophoresis is based on the electric charge of an analyte, whereas DEP is based on an induced dipole inside an analyte. Accompanying the development of microfluidics, electrophoresis can be implemented within a narrow capillary, named capillary electrophoresis (CE), and be integrated to the microfluidic chip. Because of a small amount of single-cell analysis, CE has been applied as a treatment before an analysis and has the advantage of concentrating the analyte for the next step in the analysis.

**Figure 2:**
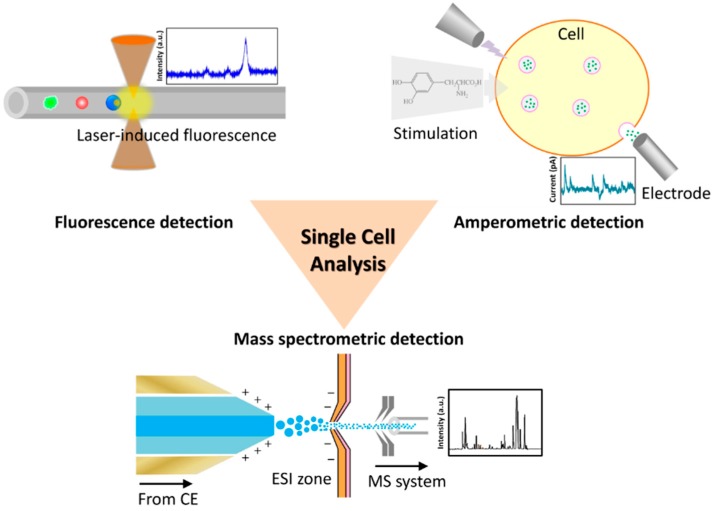
Schematic diagram of major approaches for the analysis of single cells with a microfluidic device.

In addition to lyse cells for CE approach, various non-invasive methods are applicable for live-cell detection. Investigation of live-cells has more restrictions than analyzing cell lysates because of the cell structure (e.g., cell membrane or nuclear membrane) that hinders the interactions between the labeling probes and targets of cells. Optical measurement is the most used non-invasive method for biological research; furthermore, fluorescence detection currently is simple and convenient for many kinds of research application because of the plentiful commercialized fluorescent probes. Fluorescent proteins, nanoparticles and quantum dots all can be utilized as the fluorescent source of probes, the specificity of which depend on the antibodies or DNA sequence (aptamers). Li *et al.* [33] developed a highly sensitive detection system for membrane protein of living single-cells by employing aptamer and enzyme assisted fluorescence amplification, which successfully carried out high throughput single-cell analysis of the low-abundance biomarker (PTK7). In what follows we introduce the major detection approaches for single-cell analysis (Figure 2).

### 3.1. Fluorescence Detection

Rough information about cellular contents can be acquired by lysing a trapped single cell and performing capillary electrophoresis, but such rough information to understand an organism in detail is limited. Capillary electrophoresis and laser-induced fluorescence (CE-LIF) provide scientists with more sub-cellular information about the interior of the cells. LIF employs a laser to excite fluorescence from a specific molecular moiety, and has been integrated with CE in a microfluidic device that provides a powerful technique with great sensitivity and a small detection limit for use in single-cell analysis. The fluorescein tagged substance is an analytical target of the LIF approach; the structure of the compound in the cell that signals transmission typically contains aromatic rings exhibiting self-fluorescence that is an effective candidate for LIF detection. Keithley *et al.* [34] developed a capillary electrophoresis system with a three-color laser-induced fluorescence detector to measure BODIPY fluorophores conjugated glycophingolipids. Three fluorophores (BODIPY-FL, BODIPY-TMR and BODIPY-650/665) were prepared in a chemoenzymatic synthesis and excited with diode-pumped solid-state lasers and a diode laser at wavelengths 473, 532 and 633 nm sequentially. Neuronal-like dPC12 cells were incubated with the fluorophores; their metabolic products were studied with the three-color CE-LIF system. Metto *et al.* [35] demonstrated an integrated microfluidic device to measure nitrogen oxide (NO) produced in single T-lymphocytes (Jurkat cells). The functions of cell transport, lysis, injection, electrophoretic separation and fluorescence detection were integrated in that microfluidic device. Two fluorescent probes, 4-amino-5-methylamino-2′,7′-difluorofluorescein diacetate (DAFFM DA) and 6-carboxyfluorescein diacetate (6-CFDA), were used to label NO and Jurkat cells sequentially. NO production in the cells can be stimulated on applying lipopolysaccharide (LPS) via inducible nitrogen oxide synthase (iNOS) in immune cells. The ultimate results of NO measurement from single-cell analysis compared well with the bulk cell level. Ban *et al.* employed a CE-LIF system to detect micro-RNA in cardiomyoblast cells [36] and lung-cancer cell lines [37]. Micro-RNA was captured via hybridization with DNA probes labeled with 6-FAM- or Cy5 in a separate sequence, and detected with a dual laser system. Their results showed that CE-LIF could be completed within 13 min and evaluated several endogenous miRNA.

### 3.2. Amperometric Detection

Although much subcellular information can be acquired via fluorescently based single-cell analysis, such as with fluorescein conjugated antibodies to capture proteins or fluorescein labeled probes to hybridize nucleic acids (DNA, mRNA), another approach that applied amperometry has been used to study specific proteins, especially in neurobiological research. Amperometry combined with capillary electrophoresis for electrochemical analysis of single cells becomes an effective tool to analyze chemical messengers (e.g., neurotransmitters, neuropeptides and neurohormones) in neurons; this method is also called capillary electrophoresis with electrochemical detection (CE-ED) [38]. The microfluidic device is integrated with an amperometric detecting system. After applying a stimulation of a trapped neuron (e.g., electric stimulation or chemical stimulation), chemical messengers in vesicles are released via exocytosis of a neuron of which the response can be measured. Moreover, the released chemical compounds can be collected concurrently in a microfluidic channel. Omiatek *et al.* [39] used this technique to measure the total vesicular content from single neuronal cells (PC12 cells), and found that a vesicle released only 40% of their transmitter load from a comparison of the single neuron measurement and a cell-free mode. They found also a phenomenon in which the release of a vesicular neurotransmitter and a hormone is not certainly all or none. In addition, the background noise decides the limit of amperometric detection, especially in a biosensor application. Larsen *et al.* [40] investigated the current noise caused by various electrode materials with varied capacitive properties, and concluded that low- capacitive materials and small electrodes have a small current noise, which benefits the design of a low-noise amperometric sensing device. Other than lowering the current noise, they investigated also the physical and electrochemical properties of poly(3,4-ethylenedioxythiophene):tosylate (PEDOT:tosylate) as a microelectrode material for neurochemical detection [41]. From the results, the capacitance of this conductive polymer is greater than of other thin-film materials, which limits the low-noise amperometric sensing, but this shortage can be overcome through sufficiently small fabrication. The neuron transmitter release of PC12 cells was measured with this chip-based device with microelectrodes made of PEDOT:tosylate that have the advantages of cheap and easy fabrication in all polymer devices.

### 3.3. Mass Spectrometric Detection

Mass spectrometer (MS) is a powerful tool to measure chemical components, whether used for qualitative or quantitative purpose, and is capable of providing an analysis with information about concentration, chemical structure and elemental composition. As the great sensitivity of a MS combined with CE has rapid separation and efficiency, the CE-MS analytical method becomes a suitable approach for single-cell analysis. The CE within a microfluidic device functions to selectively collect components of interest in a single cell and to concentrate the components before analyzing via a MS; the CE processed sampling approach could significantly simplify analyses entering a MS, in order to achieve a reduction of noisy signals from undesired components via the mass spectrometric analysis. With a period of CE-MS technological development, various instruments have been used for sample-ionization interfacing between a CE and a MS, such as electrospray ionization (ESI), matrix-assisted laser desorption or ionization (MALDI), laser-ablation electrospray ionization (LAESI) or inductively coupled plasma (ICP). A compatible ionizing interface is important because it affects the sensitivity of CE-MS [42]. To date, ESI and MALDI are the most popular methods for a sample–ionizing interface, but they both have advantages and disadvantages. For instance, ESI is suitable for online interfacing of a microfluidic device to a MS because of the compatible small flow rate [43]. ESI generates highly charged ions directly from a micro-fluid, which assists easy coupling between a CE and a MS, but ion suppression due to the large concentration of salts within samples becomes a challenge of analysis. In contrast, MALDI has a greater tolerance to salts, but matrix ions of MALDI limit the spectral analysis to molecular mass less than 500 Da. Aerts *et al.* [44] applied entire-cell patch-clamp recording and a CE-MS to establish the cytoplasm metabolomics of a single neuron. The physiological activity of glutamatergic thalamocortical neurons of a rat were recorded via the entire-cell patch-clamp technique, of which the metabolomics were analyzed via a CE-MS. Approximately 60 metabolites were detected and determined via a CE-MS from a tiny volume (~3 pL) of neuronal cytoplasm. This combined technique provides a new measurement tool to study the changes of cellular metabolome-related neuronal activity. Mellors *et al.* [45] developed an integrated microfluidic device that used a CE and an ESI-MS to analyze single cells automatically in real time. Human erythrocytes were lysed with this microfluidic device, of which the components were separated with the function of CE. The separated components were then ionized via an electrospray emitter and characterized in a MS. This device can verify the heme group and subunits of hemoglobin from individual erythrocytes with a throughput rate 12 cells per minute. The sensitivity of this device can ultimately be improved in optimizing the channel dimensions to decrease the possible flow rate. Smith *et al.* [46] developed an ESI-MS- integrated droplet-based microfluidic device for study of single cells with high throughput. Four populations of droplets, containing cytochrome C, α-chymotrypsinogen A, carbonic anhydrase or chicken lysozyme, were generated with a surfactant-stabilized reagent in a microfluidic device. The droplets containing sub-femtomolar quantities of an analyte were collected and then re-injected into the main channel of a microfluidic device for further characterization via an ESI-MS. This technique provides an alternative approach for analysis of single cells with high throughput.

## 4. Application Summary of Single-Cell Analysis

To date, microfluidic-based single-cell analysis has been applied in cellular research of diverse aspects and with multiple functions, for example, polymerase chain reaction (PCR) for gene analysis or cell differentiation for regenerative medicine. As mentioned at the beginning of this review, single-cell biology begins to investigate the functional mechanism of an organism from a single cell, the goal of which is the same as omic biology and provides more information about cellular heterogeneity. Many data of single-cell analysis can now be catalogued as single-cell genomics, single-cell transcriptomics and single-cell proteomics. Single-cell genomics can be established using single-cell qPCR [47], nanopore-based DNA sequencing [48] *etc.*, and provides researchers a complete understanding in order to verify whether a specific gene is constantly expressed in the same cell population or selectively expressed. Single-cell transcriptomics is thus generally established using a reverse transcriptase polymerase chain reaction (RT-PCR) [49,50], real-time PCR [51] or transcriptome sequencing (RNA-Seq) [52]. The study of single-cell transcriptomics provides intracellular variability of RNA profiles under varied environmental conditions. Compared with the omic biology of nucleic-acid-based methods, the construction of single-cell proteomics is much more difficult than single-cell genomics and transcriptomics because the small amount of protein in a single cell requires highly sensitive detection. A CE-integrated microfluidic device combined with MS-related techniques enables the convenient establishment of single-cell proteomics. The single-cell proteomics helps researchers in studying diverse post-translational modifications [53], translocations [54], and activity-correlating protein conformations [55]. Besides the above mentioned omic biology, epigenomics currently is a frontier in single-cell analysis [56]. Epigenomics investigates epigenetic modulations (e.g., DNA methylation or histone modification) of DNA, which is heritable and affects gene expressions in each single cell, although these single-cells have the same DNA sequence. Various bio-techniques now can be applied in establishing epigenomics [57], such as Chromatin immunoprecipitation followed by sequencing (ChIP-seq) [58] or bisulfite sequencing (BS-seq) [59], which have been used for locating histone modification.

Although the literature about microfluidic-based single-cell analysis is enormous, the contributions that matter most are in fundamental cellular research to provide new insights into the mechanisms of life. Single-cell analysis applications are now moving in the direction of diagnostic use and personalized medicine. The detection of circulating tumor cells (CTC) is a useful example of using a microfluidic device for diagnostic use. CTC are generated on metastasis of a primary tumor and are found in the peripheral blood from cancer patients. The variant number of CTC indicates the severity of the cancer and reducing it represents success of cancer therapies. The challenge of CTC detection is that CTC are rare in a heterogeneous sample (blood); high throughput and sensitive techniques are needed. A microfluidic device thus becomes an ideal tool to capture, separate, count and further analyze CTC efficiently [60,61,62,63]. Drug discovery for personalized medicine is also a prominent issue within single-cell analysis, to understand the mechanism of a drug-treated single cell, which exhibits a complicated response (*i.e.*, gene expression, metabolic alteration). The single-cell transcriptome analysis can provide a variation of RNA profiles after a drug treatment; the proteomic analysis can rapidly screen the candidates of antigen-specific antibodies for potential drug use [64].

## 5. Conclusions

In summary, various techniques are now available for the analysis of a single cell; with the aid of these techniques, many biological questions can be answered. A microfluidic device is perhaps now a suitable technique for single-cell analysis because a microfluidic system can be manipulated with high throughput, and the amount of sample from a single cell is limited. A microfluidic device might, however, be replaced with the further development of tools through the future efforts of physicists and engineers to answer other interesting biological questions. Although devices ready to use for diagnosis at a point of care are rare at present, most techniques have been applied in basic research. We believe that from the combination of the versatile design of microfluidic devices, flexible choice of analytical technique and increased knowledge, a portable device for personalized medical use can ultimately emerge.
